# Spatial allocation of anthropogenic carbon dioxide emission statistics data fusing multi-source data based on Bayesian network

**DOI:** 10.1038/s41598-021-93456-6

**Published:** 2021-09-13

**Authors:** Jianbin Tao, XiangBing Kong

**Affiliations:** 1 Key Laboratory for Geographical Process Analysis & Simulation of Hubei Province/School of Urban and Environmental Sciences, Central China Normal University, NO. 152 Luoyu Road, Wuhan, 430079 China; 2 Yellow River Institute of Hydraulic Research, Zhengzhou, 450003 Henan China

**Keywords:** Climate sciences, Environmental sciences

## Abstract

A gridded social-economic data is essential for geoscience analysis and multidisciplinary application. Spatial allocation of carbon dioxide statistics data is an important issue in the context of global climate change, which involves the carbon emissions accounting and decomposition of responsibility for carbon emission reductions. In this research a new spatial allocation method for non-point source anthropogenic carbon dioxide emissions (ACDE) fusing multi-source data using Bayesian Network (BN) was introduced. In addition to common-used DMSP (Defense Meteorological Satellite Program), PD (population density) and GDP (Gross Domestic Production) data, the land cover and vegetation data was imported into the model as prior knowledge to optimize the model fitting. The prior knowledge here was based on the understanding that ACDE was dominated by human activities and has strong correlations with land cover and vegetation conditions. A 1 km gridded ACDE map integrated emissions form point-source and non-point source was generated and validated. The model predicts ACDE with high accuracies and great improvement can be observed when fusing land cover and vegetation as prior knowledge. The model can achieve successful statistics data downscaling on national scale provided adequate sample data are available, offering a novel method for ACDE accounting in China.

## Introduction

The increasing greenhouse gas concentrations in the atmosphere and its resulting global warming has become the most important environmental issues^1^ that affect the world's sustainable development^2^ and is increasingly becoming the focus of global change research. Global warming is mainly due to the greenhouse effect caused by carbon dioxide, methane, etc., largely as a result of human activities over the past 50 years^3^. Carbon dioxide (CO_2_) is the main source of anthropogenic greenhouse gas and the increasing of greenhouse effect caused by CO_2_ now accounts for two-thirds of the total increasing^3^. The latest atmospheric carbon dioxide concentration released by Mauna Loa Observatory (Hawaii, USA) was high as 410 parts per million^4^. Anthropogenic Carbon Dioxide Emission (ACDE), or CO_2_ emissions from fossil fuel consumption, is the result of human activities and an important indicator for decomposition of responsibility for emission reduction.

Since 2000 the average annual growth rate of ACDE in China was around 10%^5^ , accounting for about 29% of the total global ACDE, and now is the world's leading carbon-emitting country^6^. China faces growing pressures when addressing climate change negotiations and greenhouse gas emission issues^7,8^.

ACDE data are usually released officially as statistics data, which involves different statistical criteria and spatial inconsistency^9^. It’s essential to decompose ACDE into pixel units and understand exactly the spatial distribution of ACDE. A unified finer spatial resolution ACDE map is proved to be crucial to climate change research and interdisciplinary research, also is of great value for China’s carbon reduction strategies on a regional or national scale^10,11^.

Many works have been done to develop ACDE maps on global, national and regional scales^12–14^. The existing researches depend heavily on nighttime lights data and population data, however there are several limitations in these spatial proxies. Ghost thought that the correlation of nighttime lights and ACDE was complicated and it’s not possible to make independent estimates of ACDE with it^13^. Raupach also thought that the correlation between nighttime lights and human activity was significant only in developed countries^15^. Oda argued that population statistics data did not explain the spatial pattern of nighttime lights well when spatial resolutions were finer than the country and state levels^12^. The limitation of nighttime lights was also reported by^16–18^.

ACDE is closely related to human activities such as all kinds of fossil fuel consumptions. Land cover is the source of ACDE, reflecting the intensity of human activities^19^ which has strong relationship with built-up areas. It has been proved that vegetation coverage has negatively correlation with impervious surfaces, the key component of built-up areas^20,21^. We can understand that high level ACDE can be observed in built-up areas, but we can’t expect high level ACDE in high-vegetation-covered areas. In other words, there are strong relationships between ACDE and land-cover types (including vegetation conditions). Therefore, my idea is that land cover and vegetation condition should be considered in the spatial allocation model.

The objective of this study is to develop a spatial allocation model fusing multi-source data using Bayesian Network (BN). The specific objectives include: (1) Building a spatial allocation model for non-point source ACDE statistical data fusing multi-source data, including DMSP (Defense Meteorological Satellite Program), PD (Population Density) and GDP (Gross Domestic Production) data; (2) Introducing prior knowledge, including the probability distribution information about ACDE form land cover and vegetation data, into the model to improve the performance of the model; (3) Spatial allocation of ACDE, providing a basis for carbon emission accounting and carbon emission reduction. The novelty of the proposed model is that, the land-cover types and vegetation conditions were fused into the model as prior knowledge to mitigate the limitations in commonly used spatial proxies. Accuracies analysis were conducted by comparing the model result with the statistical data and ACDE products data at provincial, city and pixel level respectively. The advantages of the model in adding land cover and vegetation data as prior knowledge are demonstrated compared with conventional methods.

## Research area and data

**Figure 1:**
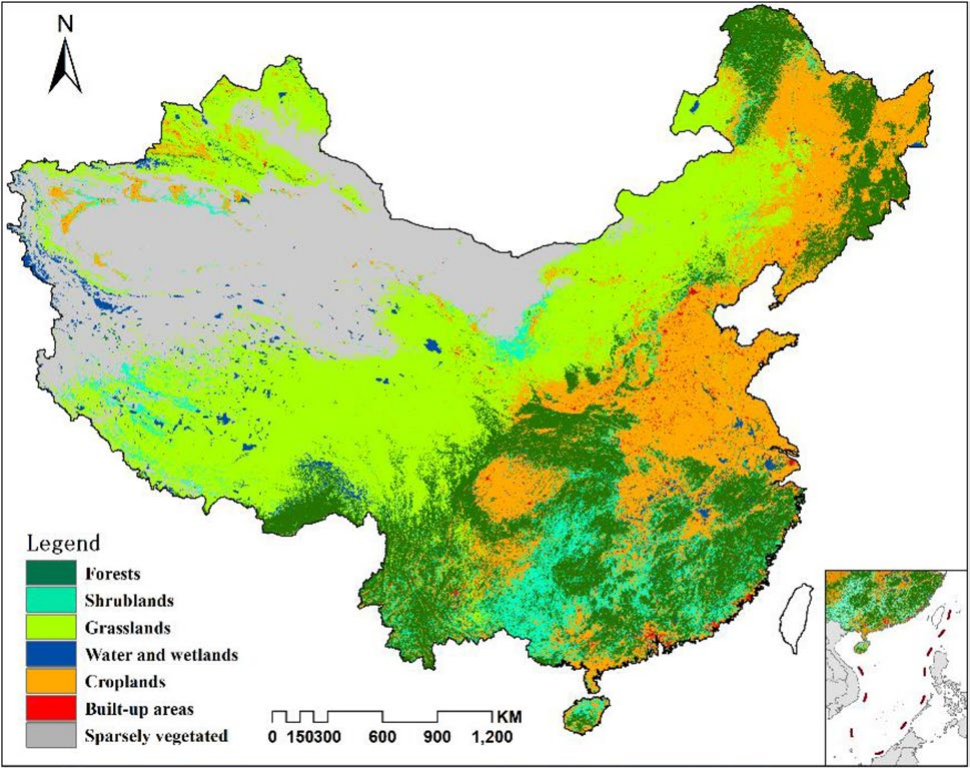
The research area and the major land-cover types in 2010 from MODIS land cover product (MCD12Q1 UMD). The fig was generated using the ArcGIS Desktop (ESRI, Inc, Version 10.2, https://desktop.arcgis.com/zh-cn/).

In this study, mainland China is selected as the research area. The major land-cover types include forests, shrublands, grasslands, water and wetlands, croplands, built-up areas and sparsely vegetated (Fig. 1). Some original IGBP (International Geosphere-Biosphere Programme) land-cover types were aggregated to obtain more general types.

**Figure 2:**
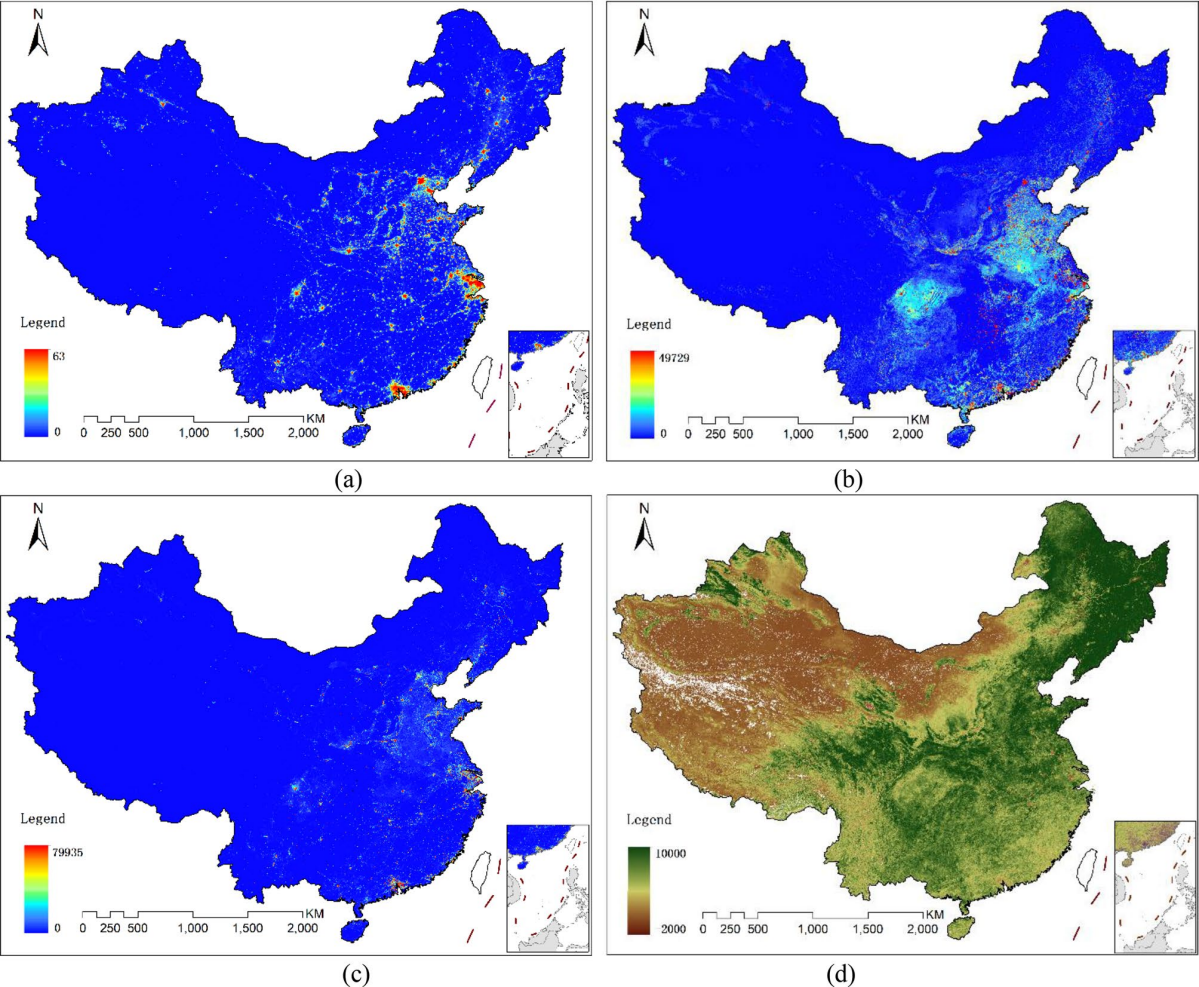
The datasets in 2010, (**a**) DMSP, (**b**) PD, (**c**) GDP, (**d**) EVI. The figs were generated using the ArcGIS Desktop (ESRI, Inc, Version 10.2, https://desktop.arcgis.com/zh-cn/).

Several data sources for 2005 and 2010 were used in this work (Fig. 2), including:

1. DMSP/OLS nighttime lights data, from the US National Oceanic and Atmospheric Administration. The data has been preprocessed to eliminate the cloud and fire etc. The data values range from 1 to 63 and has a spatial resolution of 0.008333 degrees^22^.
2. PD and GDP data. The PD and GDP data were 1 km resolution raster data, which were obtained through spatial allocation based on PD and GDP statistics data (The data was source from National Earth System Science Data Sharing Infrastructure of China (http://www.geodata.cn)). The PD and GDP statistics data were also extracted from the statistical yearbook which was used for modeling their relationship with ACDE data at provincial level.
3. Land-cover data. MCD12Q1, the global 1 km land-cover MODIS products, was selected as land-cover data. MCD12Q1 contains five land cover classification systems, within which IGBP system^23^ are commonly used.
4. Vegetation data. The vegetation data was EVI (Enhanced Vegetation Index) from the MOD13A2 v006 data, which was sourced from EOS/Terra Satellite. The products were composites synthesized over 16 days^24^ and covered years 2005 and 2010. The Savitzky-Golay algorithm was used to filter and reconstruct the time-series EVI data^25^ by referencing the pixel reliability data layer.
5. ACDE statistical data. The energy consumption statistical data of year 2005 and 2010 covering 30 provinces or municipalities (excluding Tibet, Taiwan, Hong Kong and Macau) were sourced from the China Energy Statistical Yearbook. The data used to calculate ACDE is from the primary energy consumption of end energy consumption in the balance sheet. The 2006 IPCC Guideline^26^ was used to calculate carbon-dioxide emissions from energy sources. Nine types of fossil energies, including coal, coke, crude oil, gasoline, kerosene, diesel oil, fuel oil, natural gas and electricity, were selected to calculate carbon emissions form energy consumption. The formula is as follows:
  1 $${A}_{C}=\frac{44}{12}\times \sum _{i=1}^{9}{K}_{i}{E}_{i}$$
  *A*_*c*_ is carbon emissions form energy consumption. ${E}_{i}$ is carbon emissions for energy *i* with unit 10^4^ t. ${K}_{i}$ is carbon emission coefficient for energy *i* with unit (10^4^ t carbon)/(10^4^ t coal equivalent). i denotes the type of energy. ${K}_{i}$ are default carbon emission coefficients from IPCC Guideline and are given in Table 1. Provincial ACDE statistical data are given in Table 2.
  Provincial ACDE statistical data are presented in Table 2.
6. Carbon dioxide emissions from point sources. The carbon dioxide emissions from point sources are calculated separately by using the power-plant database CARMA (Carbon Monitoring and Action, http://carma.org). All power plants that use fossil fuels were selected from the database and their emissions were recorded. All power plants located in mainland China were reviewed using Google Earth images. Those power plants that cannot match with the locations will be included in non-point source.

**Table 1:** Carbon emission coefficients for different energy types.

Energy types	Convertion to coal equivalent (t coal equivalent) (t^−1^)	Carbon emission coefficients (10^4^ t coal) (10^4^ t coal equivalent)^−1^
Coal	0.7143	0.7559
Coke	0.9714	0.855
Crude oil	1.4286	0.5857
Gasoline	1.4714	0.5538
Kerosene	1.4714	0.5714
Diesel oil	1.4571	0.5921
Fuel oil	1.4286	0.6185
Natural gas	1.33	0.4483
Electricity	0.345	0.272

^*^Conversion coefficient for natural gas is t/ten thousand m^3^. Conversion coefficient for electricity is t/ten thousand Wh.

**Table 2:** Provincial ACDE statistical data (ten thousand tons).

Provinces	2005	2010	Provinces	2005	2010
Beijing	3211.23	3488.87	Henan	11940.63	17032.00
Tianjin	3460.45	5077.85	Hubei	6798.00	10142.17
Heibei	16377.65	22951.56	Hunan	6094.49	8269.43
Shanxi	16121.15	18925.19	Guangdong	10841.27	15923.43
Inner Mongolia	8838.09	16948.17	Guangxi	2804.41	4876.04
Liaoning	13708.68	18921.23	Hainan	344.00	1351.58
Jilin	4897.36	6996.86	Chongqing	2846.83	4160.74
Heilongjiang	6903.82	9521.93	Sichuan	6036.17	8853.56
Shanghai	6372.66	7357.40	Guizhou	4679.64	6581.40
Jiangsu	13544.28	18681.41	Yunnan	5005.58	6801.31
Zhejiang	8443.77	11974.65	Shaanxi	4968.40	9370.20
Anhui	5575.45	8856.60	Gansu	3604.74	4792.75
Fujian	3730.64	6268.16	Qinghai	703.74	1043.99
Jiangxi	3325.59	4889.76	Ningxia	2055.82	3613.54
Shandong	20118.87	30514.86	Xinjiang	4014.35	7362.58

^*^Data for Tibet, Taiwan, Hongkong and Macau are missing.

All the raster datasets were re-projected into the same coordinate system (WGS 1984), and re-sampled to 1 km grid using nearest neighbor method. Zonal statistics function in ArcGIS was used to get the mean and sum value of all variables, which were used as case values for modeling and validation in provincial and city level.

## Results

The experimental analyses were conducted from two aspects: model calibration and accuracy validation. The performance of two models, the model fusing multi-source data (Model 1), and the model adding land cover and vegetation data as prior knowledge (Model 2), was compared.

### Model calibration

To test the prediction success rate of the model, the logarithmic loss, quadratic loss and spherical payoff measures^27^ were calculated to evaluate the performance of the models by using the sampling ACDE values as reference values. Their equations are^28^:

2 $$ {\text{Logarithmic~}}\,{\text{loss}} = M\left( { - {\text{ln~}}S} \right) $$

3 $$ {\text{Quadratic}}\,{\text{loss}} = M\left( {1 - 2S + \mathop \sum \limits_{{j = 1}}^{n} P_{j}^{2} } \right) $$

4 $$ {\text{Spherical}}\,{\text{payoff}} = M\left( {\frac{S}{{\mathop \sum \nolimits_{{j = 1}}^{n} P_{j}^{2} }}} \right) $$

where *M* represents the mean probability value of a given state, *S* is the probability of the correct state for a given class, *P*_*j*_ is the probability for class *j* and *n* is the number of states.

I took 2010 data as training data and 2005 data as validation data. The root mean square error (RMSE) and the relative error rate were used to test the accuracy of the model outputs. The relative error rates were 18.75% and 9.68% for the two models respectively (Table 3).

**Table 3:** Overall accuracy measures of the models.

	Relative error (%)	Logarithmic loss	Quadratic loss	Spherical payoff
Model 1	18.75	2.16	0.31	0.82
Model 2	9.68	0.17	0.12	0.93

### Accuracy validation

A quantitative accuracy comparison with statistical data and the products from FFDAS, EDGAR (the Emission Database for Global Atmospheric Research) and ODIAC (Open-Data Inventory for Anthropogenic Carbon dioxide) were performed. EDGAR is a global-covered 10 km × 10 km resolution emission inventory data developed by EC-JRC/PBL^29^ by using point source and road network data in addition to population data. FFDAS is a global fossil fuel CO_2_ emissions inventory developed by Rayner et al.^30^ by assimilating nightlights data together with population data. ODIAC is a global high-resolution emission data product for carbon dioxide emissions, originally developed under the Greenhouse gas Observing SATellite (GOSAT) project at the National Institute for Environmental Studies (NIES), Japan (http://odiac.org/).

The estimated ACDE were analyzed and compared at provincial, city and pixel level respectively. On provincial scale, the ACDE statistical data in 2005 were used as reference. At city level, FFDAS ACDE product data in 2005 was used as reference to compare the precision of the estimated ACDE. Furthermore, the spatial distributions and qualitative evaluations of the estimated ACDE on local scale comparing with ACDE products data were carried out.

#### Provincial level

**Figure 3:**
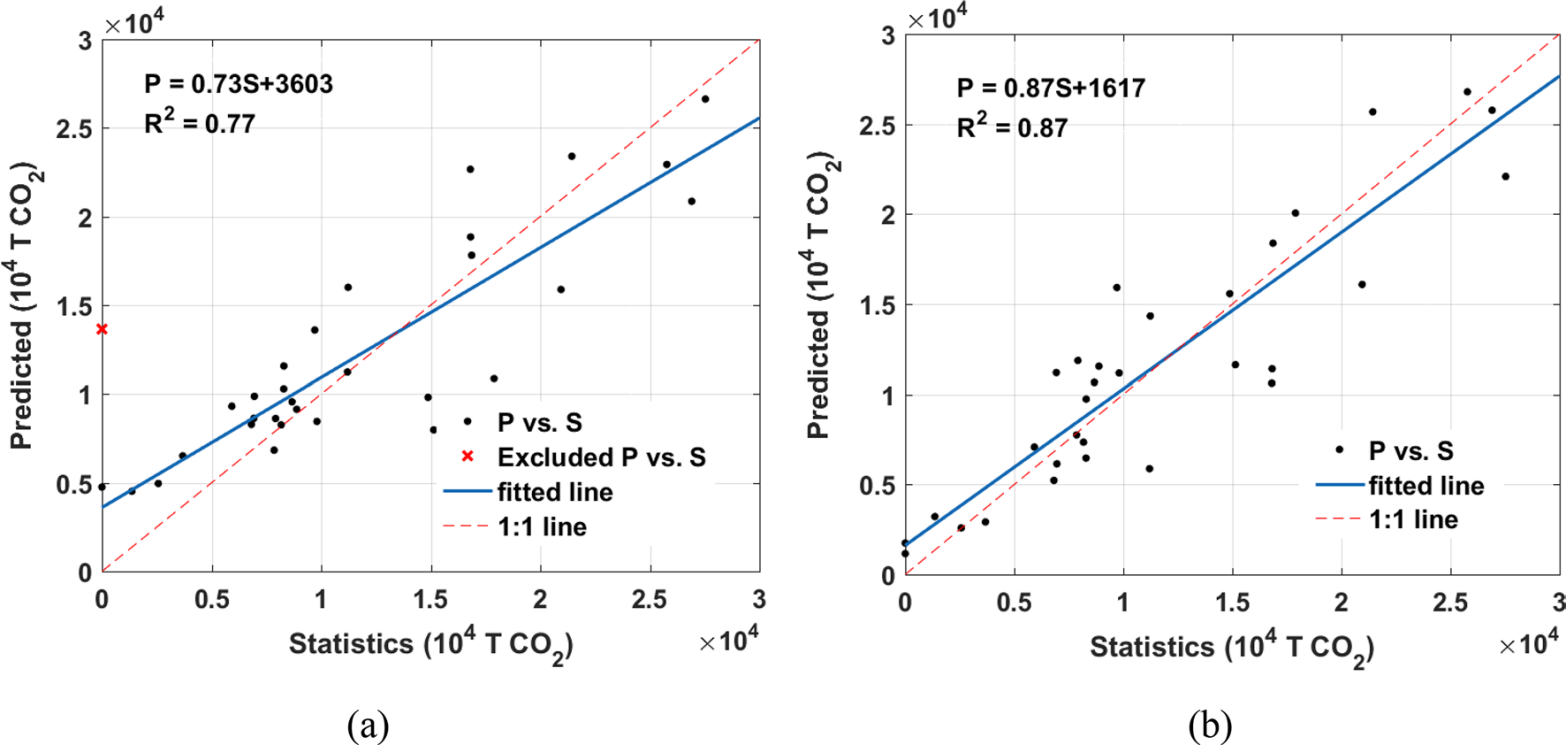
Trend line of the predicted (‘P’ for short) and the statistics (‘S’ for short) data at provincial level, (**a**) Model 1, (**b**) Model 2. The figs were generated using the MATLAB software package (The MathWorks, Version 2013, https://www.mathworks.com). The predicted data was summarized to provincial level using zonal statistics tool in ArcGIS. Two outliers were excluded because of the missing value in statistic data.

The scatter diagrams of the result predicted by the BN model with or without prior knowledge versus statistics data at provincial level are plotted in Fig. 3.

The proposed BN model predicted ACDE with higher accuracies at provincial level. Furthermore, the Model 2 predicted ACDE better than Model 1 with R^2^ of 0.87 because of its full consideration of land cover and vegetation coverage as prior knowledge.

#### City level

Although built-up areas cover a small percentage of earth surface, they influence vast areas due to the massive energy demands^31^, and are main source of ACDE^32^. So we can believe that the accuracy at city level can explain most variations of the BN modeled result.

**Figure 4:**
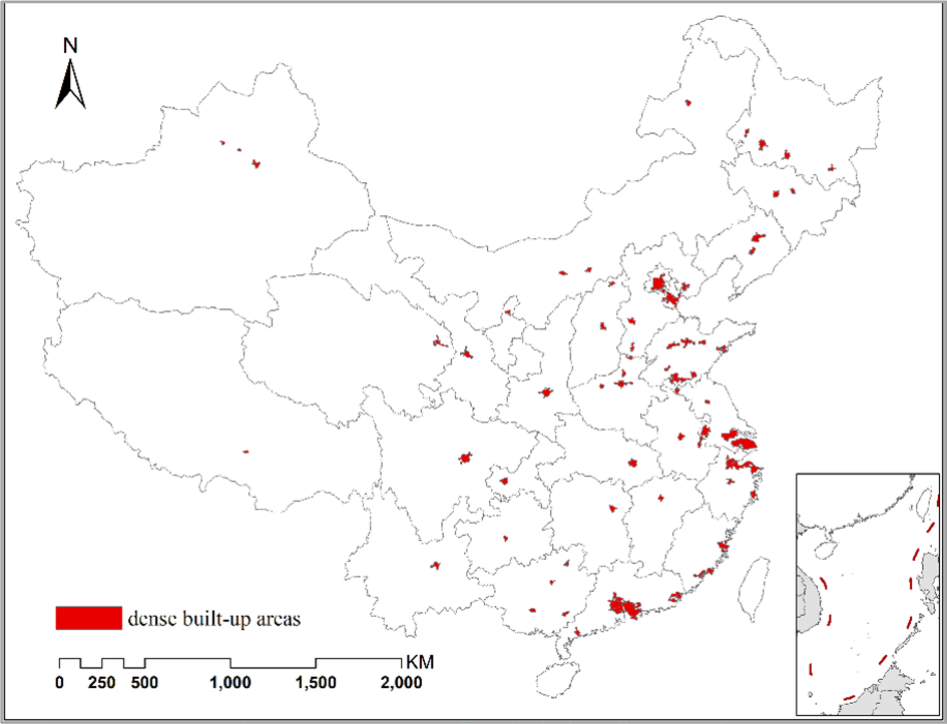
The distribution of the selected homogeneous areas covering dense built-up areas. The fig was generated using the ArcGIS Desktop (ESRI, Inc, Version 10.2, https://desktop.arcgis.com/zh-cn/).

At city level, the statistic units are not administrative region but are image blocks generated through segmentation of nighttime lights data. Multiresolution segmentation was conducted on DMSP data to generate homogeneous areas using Definiens Developer 7.0. The scale was set to 120, shape *vs* color was set to 0.7:0.3, and compactness *vs* smoothness was set to 0.9:0.1. Those homogeneous areas with mean value greater 10 and sum value greater than 10,000 were selected and merged. These areas cover most of the built-up areas, and also represent most prefectures-above-level cities (Fig. 4). In this sense, this level is a selected city level.

**Figure 5:**
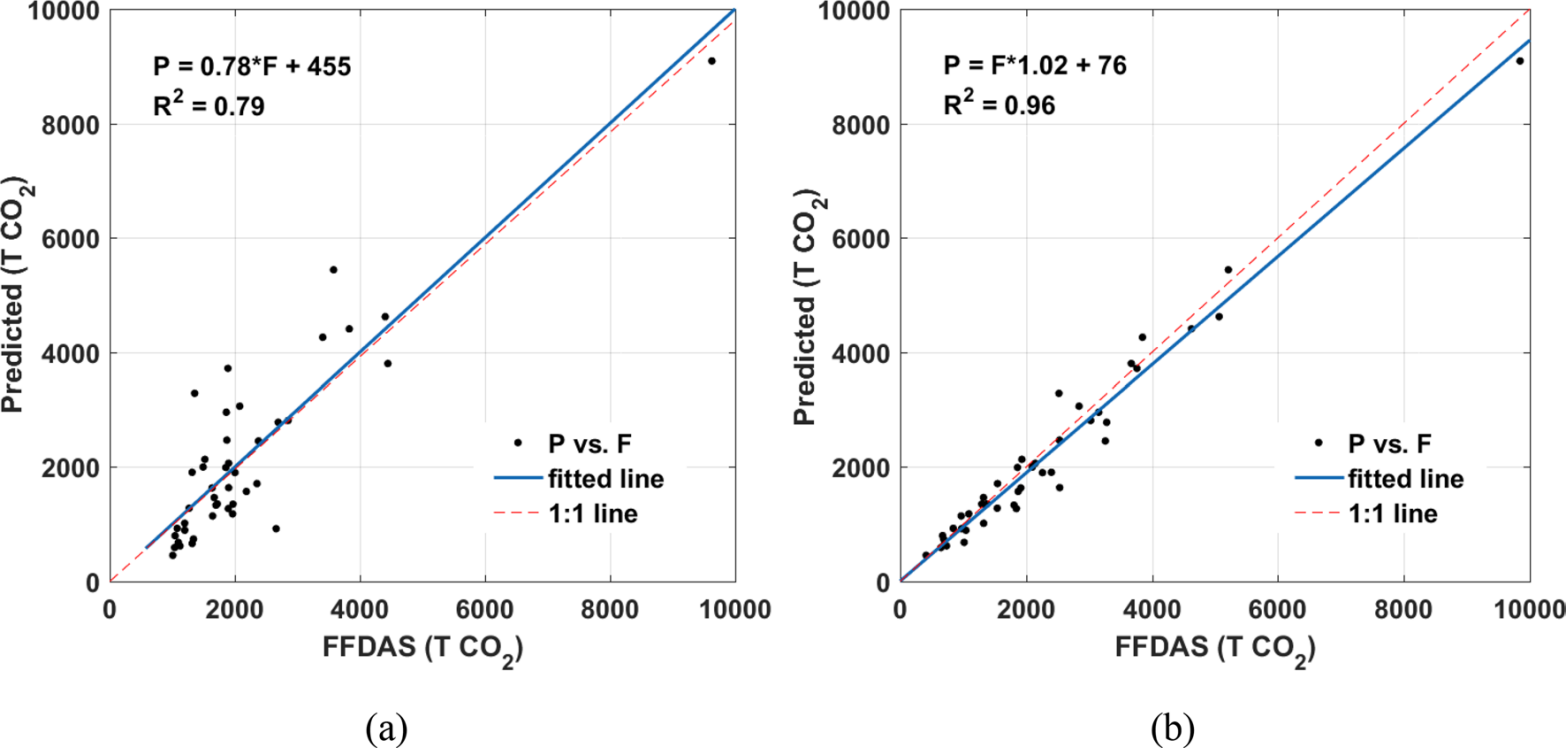
Trend line of the predicted (‘P’ for short) and FFDAS (‘F’ for short) data at selected city level, (**a**) Model 1, (**b**) Model 2. The figs were generated using the MATLAB software package (The MathWorks, Version 2013, https://www.mathworks.com).

The precisions of Model 1 and Model 2 are evaluated by comparing with FFDAS data at city level (Fig. 5). Since it’s hard to collect ACDE statistics data at city level, FFDAS data was used as reference and summarized to city level (considering the borders I defined through image segmentation), although it also has substantial error.

The R^2^ of Model 2 reaches 0.96 at confidence level of 95%. Great improvement can be observed from the Model 2 at city level when fusing the land cover and vegetation as prior knowledge.

#### Pixel level

**Figure 6:**
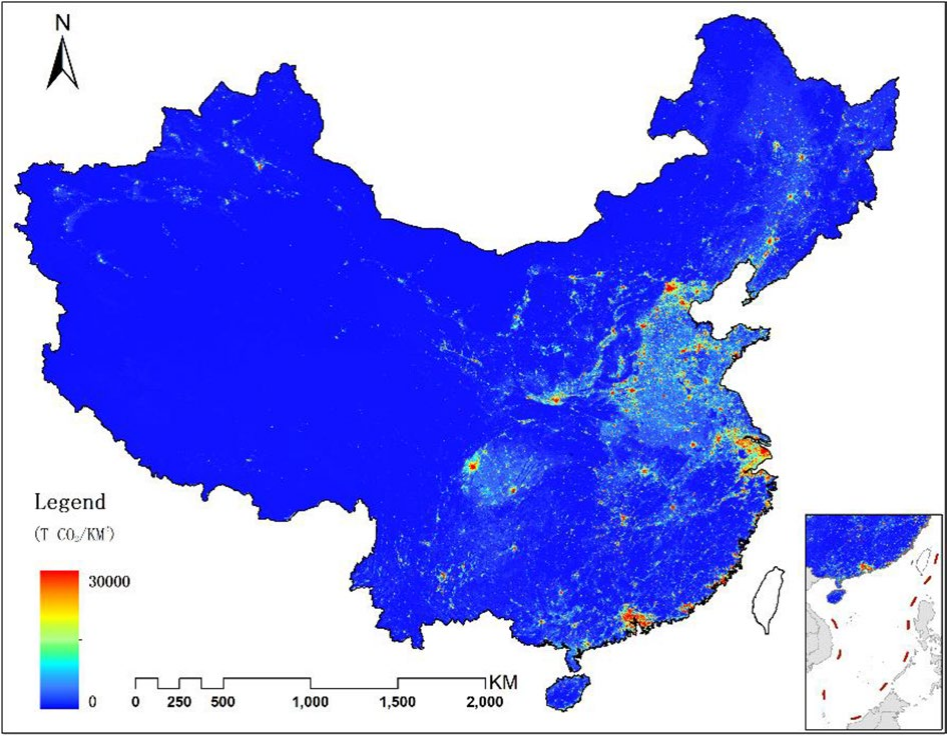
BN modeled ACDE map of mainland China in 2010. The fig was generated using the ArcGIS Desktop (ESRI, Inc, Version 10.2, https://desktop.arcgis.com/zh-cn/).

The map of the modeled ACDE was shown in Fig. 6. The map clearly shows the high ACDE emission areas in China, covering the major metropolitan areas including Beijing-Tianjin-Tangshan, Yangtze River Delta, Pearl River Delta, North China Plain, Central Urban Cluster and Sichuan Basin.

**Figure 7:**
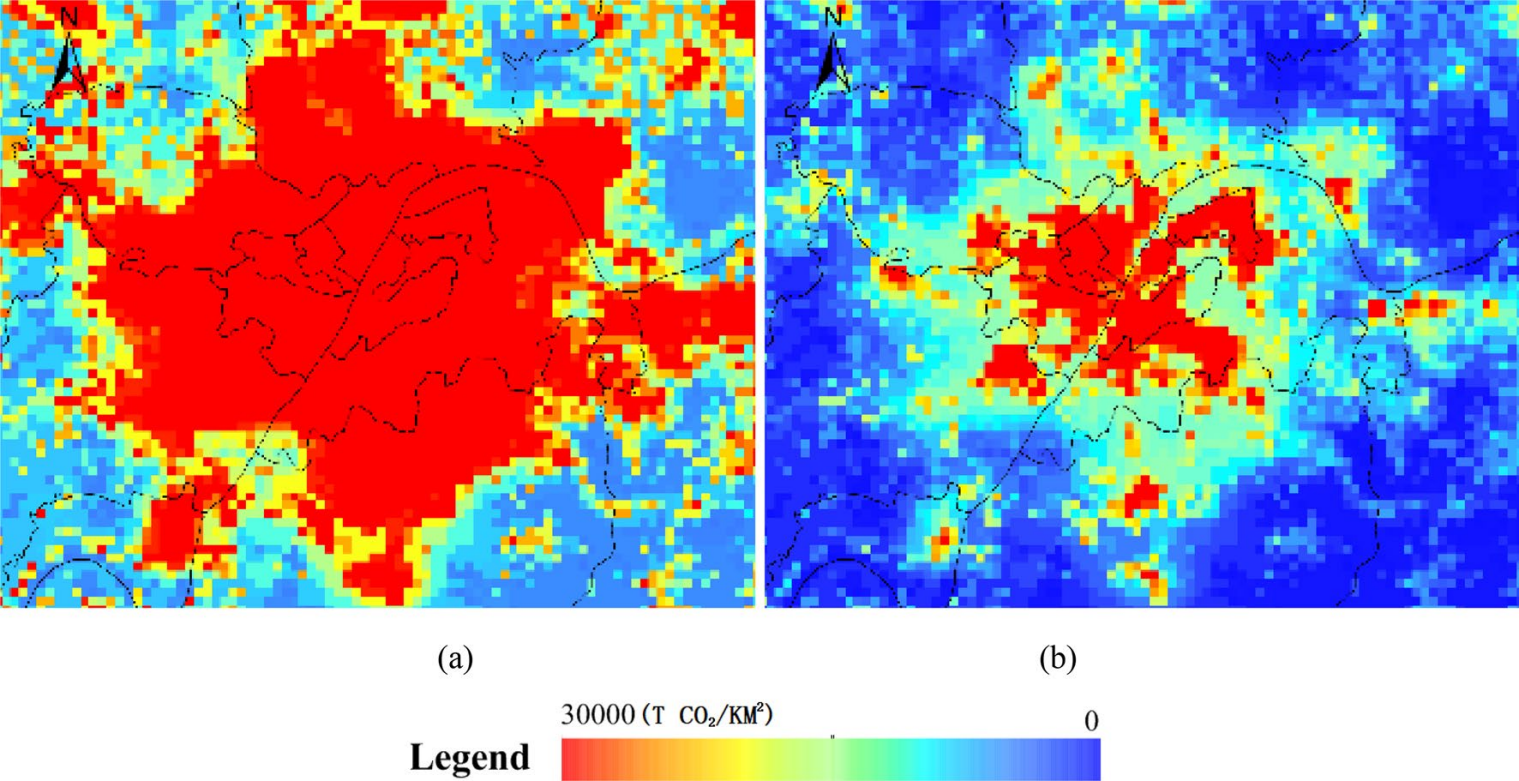
ACDE maps of major metropolitan areas in 2010, (**a**) Model 1, (**b**) Model 2. The figs were generated using the ArcGIS Desktop (ESRI, Inc, Version 10.2, https://desktop.arcgis.com/zh-cn/).

On local scale, abundant details can be observed in ACDE map derived by Model 2, especially great variations in high-density built-up areas (Fig. 7, take Wuhan urban areas as an example).

**Figure 8:**
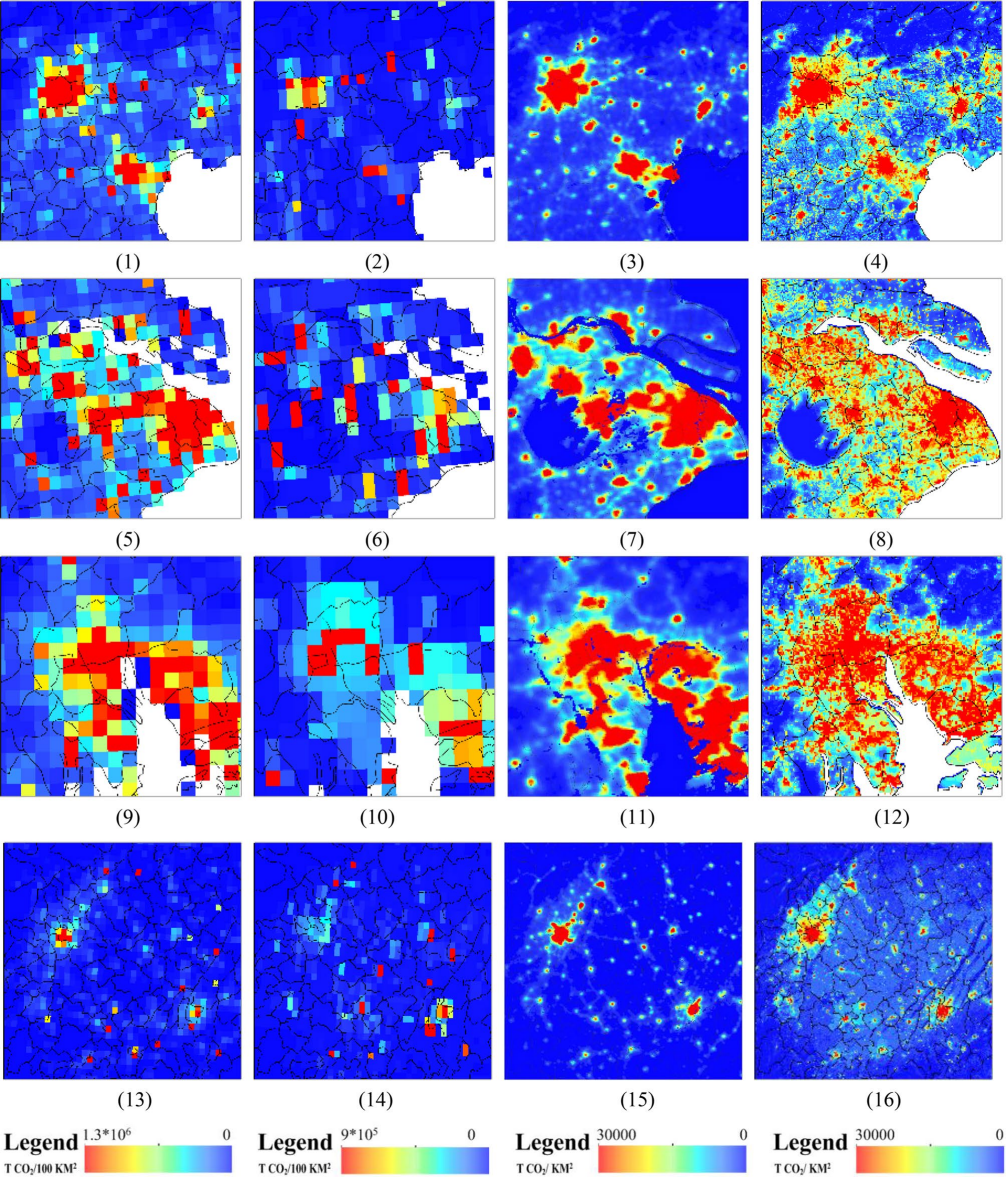
BN modeled ACDE maps comparing with ACDE products in major metropolitan areas of mainland China in 2010, (1) Beijing-Tianjin-Tangshan FFDAS, (2) Beijing-Tianjin-Tangshan EDGAR, (3) Beijing-Tianjin-Tangshan ODIAC, (4) Beijing-Tianjin-Tangshan BN modeled, (5) Yangtze River delta FFDAS, (6) Yangtze River delta EDGAR, (7) Yangtze River delta ODIAC, (8) Yangtze River delta BN modeled, (9) Pearl River delta FFDAS, (10) Pearl River delta EDGAR, (11) Pearl River delta ODIAC, (12) Pearl River delta BN modeled, (13) Sichuan Basin FFDAS, (14) Sichuan Basin EDGAR, (15) Sichuan Basin ODIAC, (16) Sichuan Basin BN modeled. The figs were generated using the ArcGIS Desktop (ESRI, Inc, Version 10.2, https://desktop.arcgis.com/zh-cn/).

The modeled ACDE was compared with ACDE products, FFDAS, EDGAR and ODIAC at pixel level (Fig. 8). The BN modeled ACDE maps in major metropolitan areas showed good spatial agreement with ACDE products, at the same time with higher spatial resolution.

## Discussions

When conducting land-surface parameter estimation, simply learning a model from a few independents without considering prior knowledge is ill-posed. The existing researches usually utilize regression method to model the relationships between spatial proxies and ACDE, depending heavily on nighttime lights data and population data^12–14,34^, without considering model’s bias caused by spatial proxies such as DMSP data. The prior knowledge that ACDE was dominated by human activities and has strong correlation with land cover and vegetation condition was usually ignored in conventional methods. The proposed model mitigated the limitations in these spatial proxies to some extent by introducing geo knowledge into earth observation, providing a new way in land-surface parameters inversion.

Predicting ACDE from multi-source data involve the ability of model to combine evidence from remote sensing observations with prior knowledge. Different from conventional statistical methods such as regression, BN can model not only conditional dependence, but also causation relationship between variables. The causal relationships between ACDE and land cover and vegetation were added as prior knowledge to the original BN model. The model structure was very simple and can be explained because the only adaption to a conventional BN model was adding a node into the model to represent the prior knowledge. The model was then locally trained and at the same time globally optimal^33^.

The proposed BN model can be applied to high accurate mapping of ACDE on national scale successfully. Furthermore, the model can be applied to any scale land-surface parameters mapping, provided the relationships between dependent variable and independent variables can be modeled by CPT and prior information about the dependent variable can be obtained.

Information loss sourced from data preprocessing method of BN is one limitation of the study. The model has relatively high accuracies in urban and built-up areas, however poor accuracy in rural areas. This is partly because the ACDE value was overestimated in rural areas, and the summation of massive rural pixels pushed up the total ACDE value. The overestimation was partly due to the discrete of variables in Netica, which cause the BN to lose statistics accuracy to some extent^35^. If the information loss caused by Netica can be avoided, the performance could be further improved.

## Conclusions

Information on the extent and spatial details of ACDE is a key requirement for understanding how human activities affect climate change. This paper reports my work on spatial allocation of ACDE statistical data in mainland China using Bayesian Network. Bayesian Network combines the robustness of probability theory with the expressiveness of graphs, offering a mechanism for handling prior knowledge in estimating land-surface parameters. A spatial allocation BN model for non-point source ACDE statistics data fusing multi-source data was developed. The BN model predicted ACDE with R-square of 0.87 at provincial level and R-square of 0.96 at city level and great improvement can be observed from the proposed model (Model 2) when fusing the land cover and vegetation as prior knowledge. The study is of great value to the development of spatial allocation method for ACDE statistics data on national scale.

The contribution of this research is that it provides a novel way of combining DMSP, PD, GDP data and land cover and vegetation data and can be applied to large-scale ACDE mapping. BN model can model not only conditional dependence between ACDE and proxies, but also causation relationship between ACDE and land covers and vegetation well. The method has the superiority of fusing land cover and vegetation data as prior knowledge into BN model, mitigating the limitations in conventional spatial proxies greatly. Conventional approaches for downscaling statistical data into grid level based on nightlight and population data have certain limitations. BN model can therefore provide a good solution to spatial allocation of ACDE statistical data.

## Methods

ACDE gridded data were estimated by combining emissions from point source and non-point source. ACDE from point source were calculated separately using the CARMA power plants database. Emissions form power plants were spatially allocated to the exact grid indicated by CARMA. Emissions from non-point source (including industrial, residential, commercial and transportation sectors) were obtained approximately by subtracting the emissions from point source, and then allocated to grids using Bayesian Network by fusing multi-source data as proxies. Finally, emissions form these two sources were integrated by summarizing at 1 km grid level.

**Figure 9:**
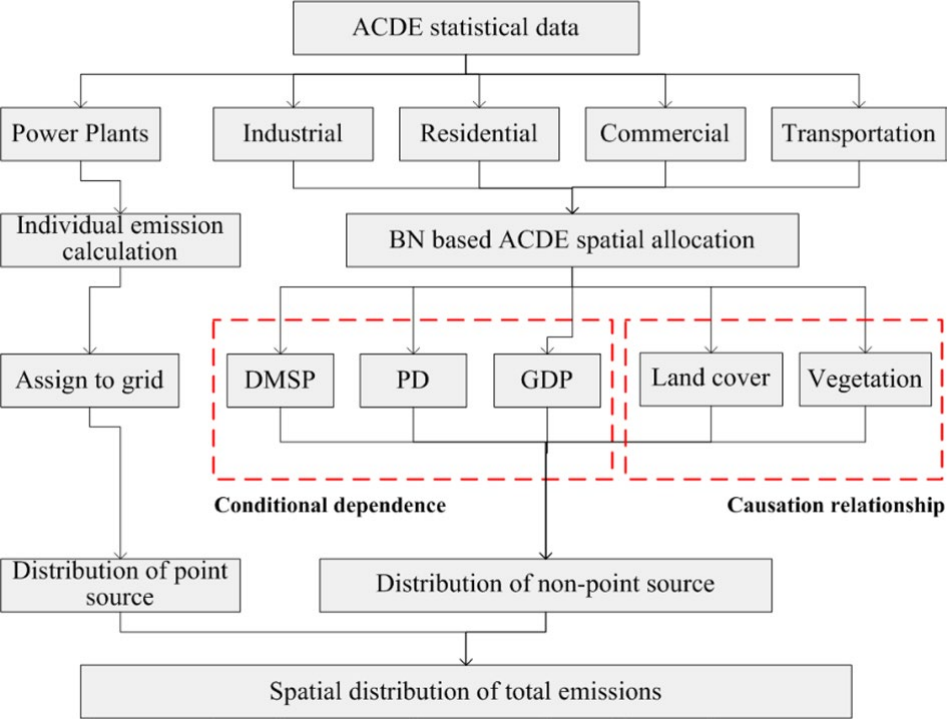
Flow chart of spatial allocation of ACDE statistical data.

By utilizing the advantages of BN, a spatial allocation model for ACDE statistics data (from non-point source) fusing multi-source data was made. Here I present the method of fusing multi-source data using BN (modeling conditional dependence between variables), and adding prior knowledge to the model (modeling causation relationship between variables). The flow chart of spatial allocation was presented in Fig. 9. The software packages used for modeling and the subsequent calibration and validation were Netica 5.02 and Matlab.

### Bayesian network

Bayesian Network is a powerful mathematical tool combing the advantages of probability theory and graph theory to reason about uncertainty in land-surface parameters estimation. A Bayesian Network (BN) is a DAG (directed acyclic graph) combined with a CPT (conditional probability table), in which each node represents a random variable and the arcs linking the nodes represent relationships between variables. In a BN the joint probability distribution of all random variables can be decomposed as the product of a series of probabilities each representing a subset of the variables. Therefore the joint probability distribution of a BN with *n* nodes can be calculated as^36^:

5 $$p\left(X\right)={\sum }_{i=1}^{n}p\left({x}_{i}|{pa}_{i}\right),$$

where ${x}_{i}$ is a random variable, ${pa}_{i}$ denotes the parent node sets of ${x}_{i}$, and $\mathrm{X}=\left\{{x}_{1},\dots ,{x}_{n}\right\}$.

Naïve Bayesian Network (NB for short) is the simplest type of Bayesian Network in which class node is the parent node of all other feature nodes and the features are assumed to be independent of each other. Therefore on the basis of conditional independence hypothesis the joint probability of all nodes is^37^:

6 $$P\left({X}_{1},{X}_{2},\dots ,{X}_{n},C\right)=P\left(C\right)\prod _{i=1}^{n}P\left({X}_{i}|C\right),$$

where *X*_1_, *X*_2_, …, *X*_*n*_ represent the features and *C* represents the class variable.

The Bayesian Network model has the advantage of having bidirectional inference capability which enables probability to be propagated forward or backward through nodes^38^.

### Fusing multi-source data

The spatial distributions of ACDE are highly correlated with human activities and land-cover conditions. As spatial proxies of ACDE, the independent variables include: (1) DMSP nighttime lights data; (2) socioeconomic data such as PD and GDP data; (3) land cover and vegetation data.

**Figure 10:**
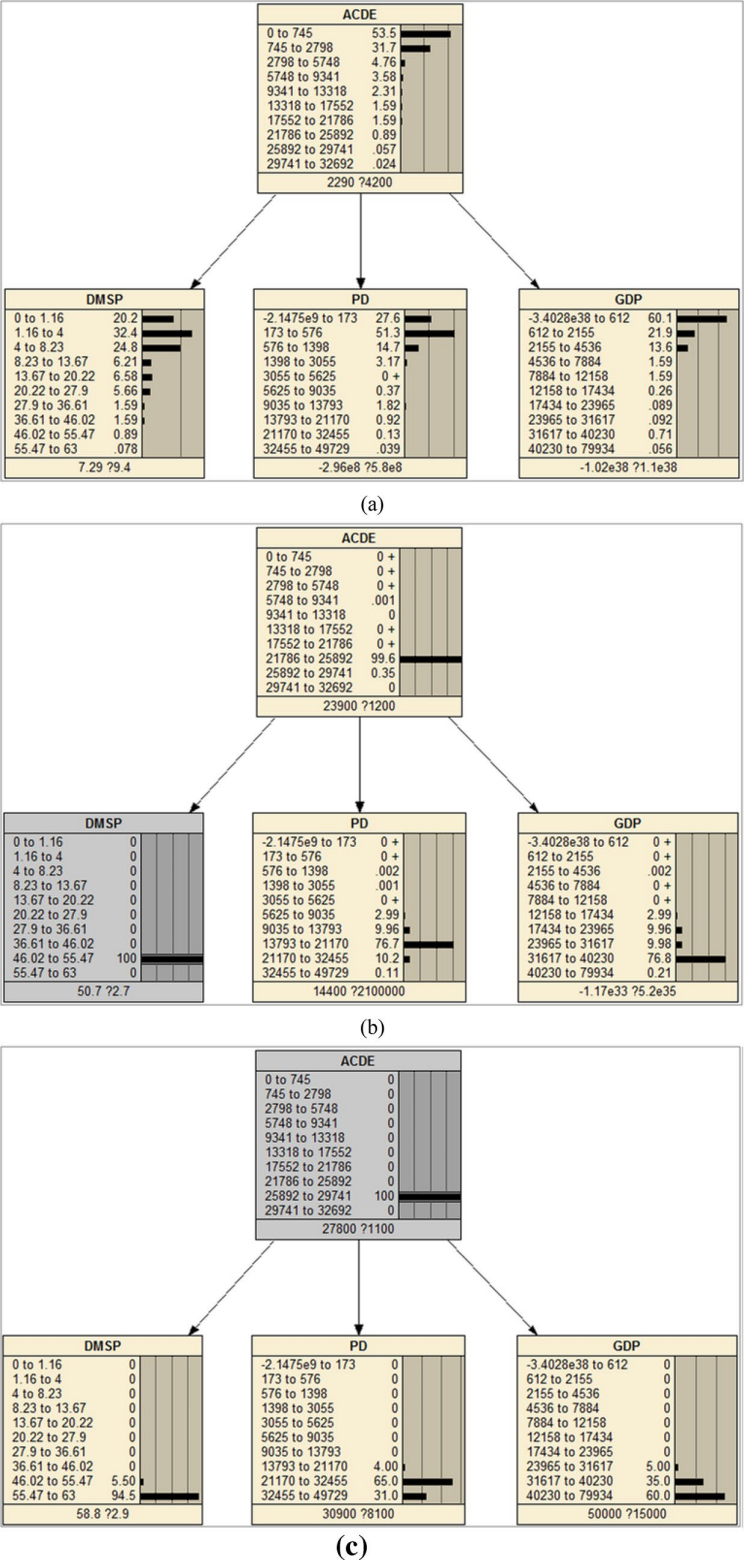
The Naïve Bayesian Networks and the CPTs of the nodes. (**a**) CPTs when learning the network, (**b**) CPTs when giving an evidence of DMSP, (**c**) CPTs when giving an evidence of ACDE. The figs were generated using the Netica software (Norsys Software Corp, Version 5.02, https://www.norsys.com/).

A Bayesian Network model was constructed to model the conditional dependence between ACDE (dependent variable) and multi-source data (independent variables). In this research, I used term variable to indicate any features. Since we know the positive relationships between DMSP, PD, GDP and ACDE, the link was added manually to construct the DAG. The initial Bayesian network was a NB which included three child nodes (Fig. 10a).

The continuous variables need to be converted to discrete quantities before any probabilistic inference because all of probabilistic inference in Netica is done with discrete tables^39^. Jenks Natural Breaks^40^ was used to discrete all the variables. After a series of tests (initializing 3–15 states) to figure out how many states can improve the overall accuracy, ten states were finally selected. The CPT of the model was estimated using the EM (expectation-maximization) algorithm. The maximum number of iterations was set to 500 to ensure convergence.

When the CPTs of each node have been determined, the network is ‘solved’^40^, as shown in Fig. 10a. The observations of the independent can then easily be interpreted by individual case. The effect of entering an evidence on one node can be examined by the response of other nodes through propagation, as illustrated in Fig. 10b, c. The rapid information propagation through the nodes make us quickly observe how conditions at one node will affect the whole network^40^. The BN is complete and can then be applied to validation and prediction of the samples after evaluation tests.

Since the relationship between independents and ACDE remain constant when change the scale from district polygon to pixel grid, the BN model can allocate ACDE statistics data to pixel level spatially. Because I can only get a continuous and complete official data at provincial level, the model was built at this level (forward), and then backward to pixel level.

In the inference stage the probability values with maximum posterior probabilities were selected as the predicted ACDE values. The Bayesian Network model can then be run on every pixel in the research area.

### Adding land cover and vegetation data as prior knowledge

Here the prior knowledge refers to the knowledge about the distribution of ACDE in addition to the probability distribution function from DMSP, GDP and PD data through training. The basis is that simply determining a model from training samples without prior knowledge is ill-posed. According to Bayes’ theorem, if *X* and *Y* are two variables, with their probabilities being *P*(*X*) and *P*(*Y*) respectively, then

7 $$P\left(X|Y\right)=P\left(Y|X\right)P(X)/P(Y)$$

where *P*(*X*|*Y*) is posterior probability (the probability of *X* given *Y*), and *P*(*Y*|*X*) is prior probability (the probability of *Y* given *X*). The prior probability is useful because it gives us important information about the predicted variable which cannot obtain from the training data.

**Figure 11:**
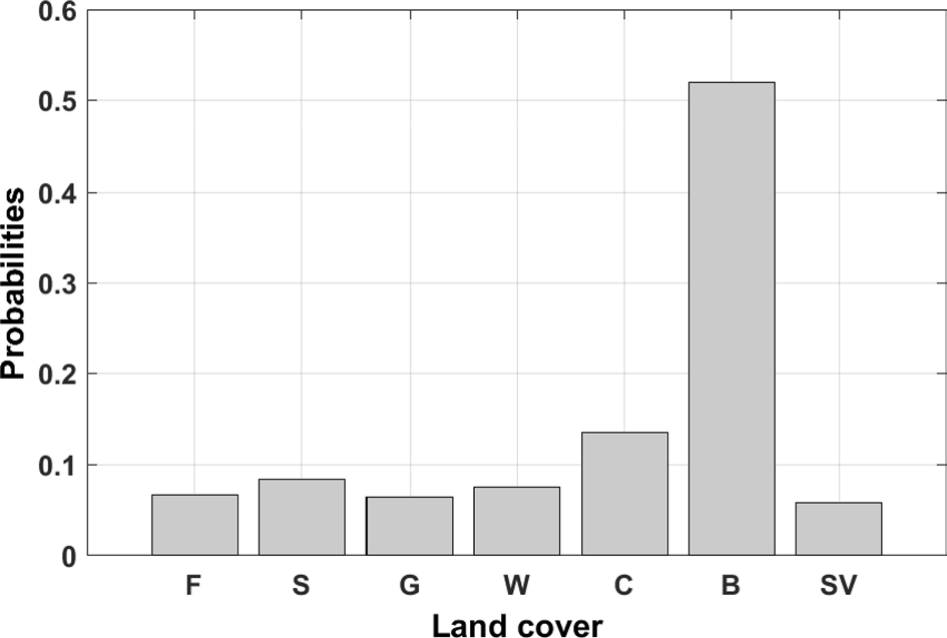
The statistics analysis of the FFDAS data compared with land-cover data. The horizontal axis indicates land-cover types: F-forests, S-shrublands, G-grasslands, W-water and wetlands, C-croplands, B-built-up areas and SV-sparsely vegetations.

Land covers document the places where human activities take place and are source of ACDE, reflecting the intensity of human activities. General speaking, ACDE usually take place in built-up areas, especially in dense urban areas. At the same time, we cannot expect significant ACDE at high-level vegetation-covered areas. To see if BN can model this causation relationship, a statistics analysis of total ACDE from FFDAS data and total built-up areas from MCD12Q1 land-cover data was conducted to explore the relationship between ACDE and land-cover types. Figure 11 shows the proportions of ACDE caused by every land-cover types. We can observe a varied proportion between them, within which built-up areas ranks top one. This percentage can be interpreted as probability of ACDE level of a pixel, given that we know the land-cover type of that pixel.

**Figure 12:**
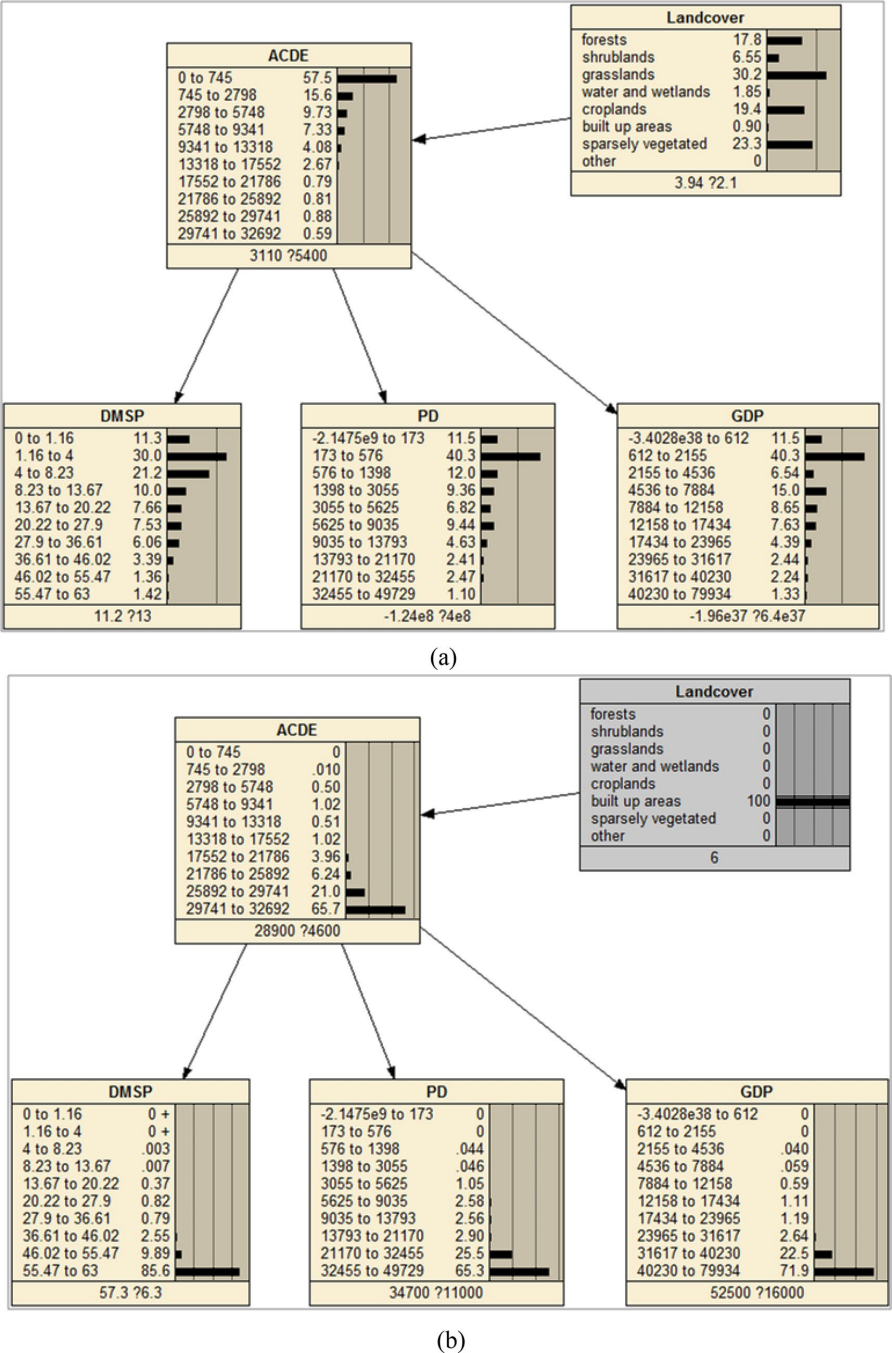
The Naïve Bayesian Network fusing land-cover data as prior knowledge. (**a**) CPTs when adding a land-cover node as the prior knowledge, (**b**) CPTs when give an evidence of land cover. The figs were generated using the Netica software (Norsys Software Corp, Version 5.02, https://www.norsys.com/).

**Figure 13:**
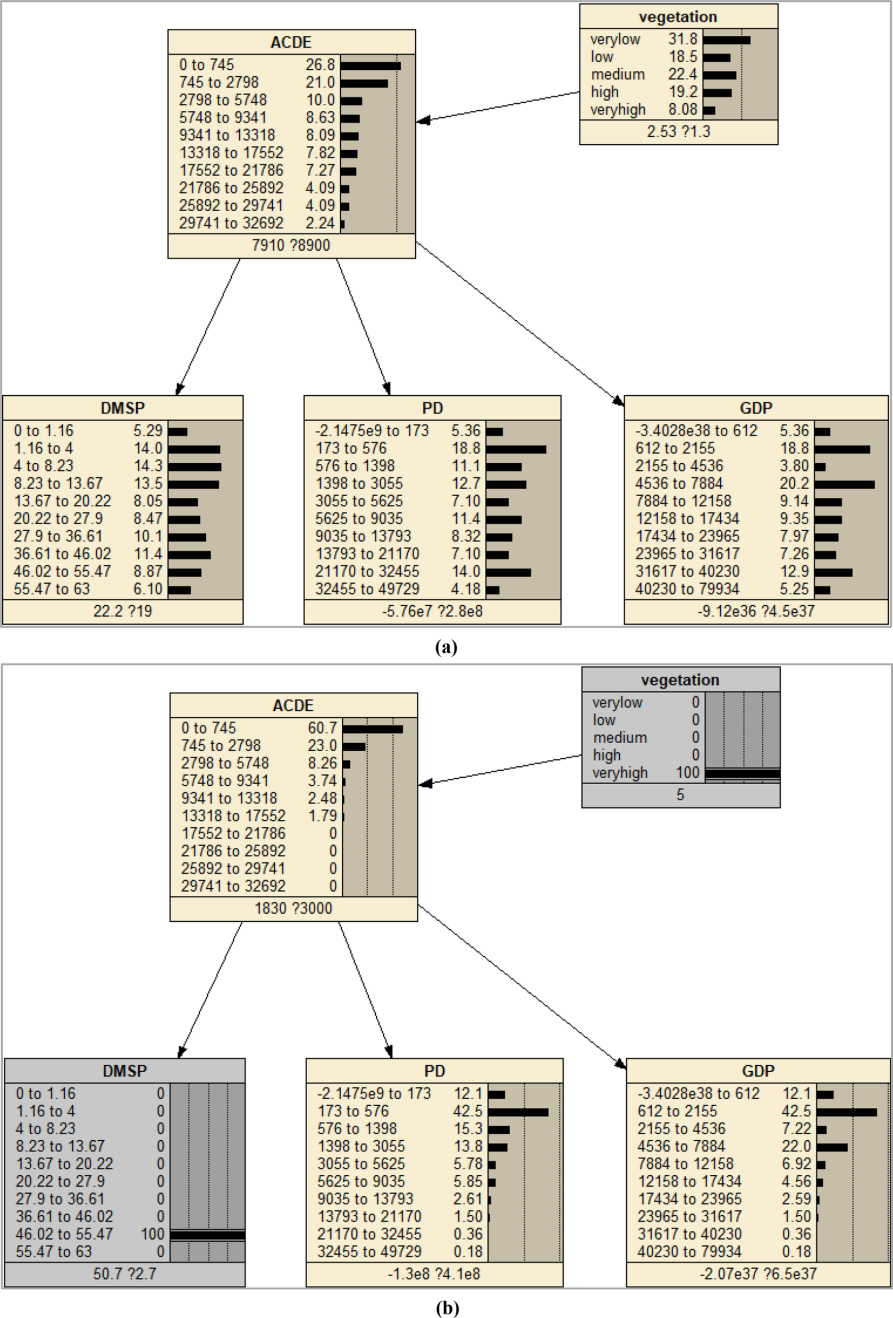
Naïve Bayesian Network fusing vegetation data as prior knowledge. (**a**) CPTs when adding a vegetation node as the prior knowledge, (**b**) CPTs when give an evidence of vegetation. The figs were generated using the Netica software (Norsys Software Corp, Version 5.02, https://www.norsys.com/).

The land cover and vegetation data were fused into the initial model (named as Model 1) as prior knowledge to generate the final model (Model 2). The original MODIS EVI data was reclassified into five classes using Jenks Natural Breaks method which represent different levels of vegetation coverage (Table 4). Unlike the training data such as nighttime lights having continuous measurement, the land cover and vegetation data is a kind of data with nominal measurement, which can’t be modeled as a natural node in BN and can’t learning CPT through training. The CPTs of land cover and vegetation nodes were input manually according to above statistics analysis. Figures 12 and 13 give the DAGs and CPTs of Model 2.

**Table 4:** Summary classes of vegetation.

EVI value	Class label	Summary class
0–1058	1	Very low
1059~2431	2	Low
2432–3632	3	Medium
3633–4961	4	High
4962–10,000	5	Very high

The CPT of land-cover node came from the statistics of FFDAS data. After adding this node, all the CPTs in other nodes update automatically. Most places have relative low level ACDE value, as well as DMSP, POP and GDP values, which can be observed from the CPTs. This is consistent with the fact that human activities and ACDE exist in relative low percent of the total land covers (Fig. 12a). If give an evidence of land cover type as built-up at 100% probability, a high level ACDE can be observed, and all the other nodes update the CPT through propagation of probabilities. If land cover tells it is built-up, it is very likely (more than 65% probability) it has high level ACDE, even if other three independents observe low values (Fig. 12b). These facts demonstrated that BN can model the causation relationship of ACDE and land covers well.

The CPT of vegetation node came from the statistics of EVI data. After adding this node, all the CPTs in other nodes update automatically (Fig. 13a). If give an evidence of vegetation condition as “very high” at 100% probability, a low level ACDE can be observed, although giving an evidence of DMSP as high at 100% probability (Fig. 13b). This seemingly contradictory can be explained by the saturation problem of DMSP in dense built-up areas. These facts demonstrated that BN can model the causation relationship of ACDE and vegetation well.
